# Supplementary material for: Characteristics and Survival Outcomes of Hepatocellular Carcinoma Developed after HCV SVR
Source: Cancers (Basel). 2021 Jul 9;13(14):3455. doi: 10.3390/cancers13143455 (PMC8306695; doi:10.3390/cancers13143455)
Supplement: Supplementary file 1 [file cancers-13-03455-s001.zip › cancers-1274704-supplementary.pdf]

Supplementary Materials: Characteristics and Survival Outcomes of Hepatocellular Carcinoma Developed After HCV SVR

Ming-Lun Yeh, Po-Cheng Liang, Pei-Chien Tsai, Shu-Chi Wang, Jennifer Leong, Eiichi Ogawa, Dae Won Jun, Cheng-Hao Tseng, Charles Landis, Yasuhito Tanaka, Chung-Feng Huang, Jun Hayashi, Yao-Chun Hsu, Jee-Fu Huang, Chia-Yen Dai, Wan-Long Chuang, Mindie H. Nguyen and Ming-Lung Yu

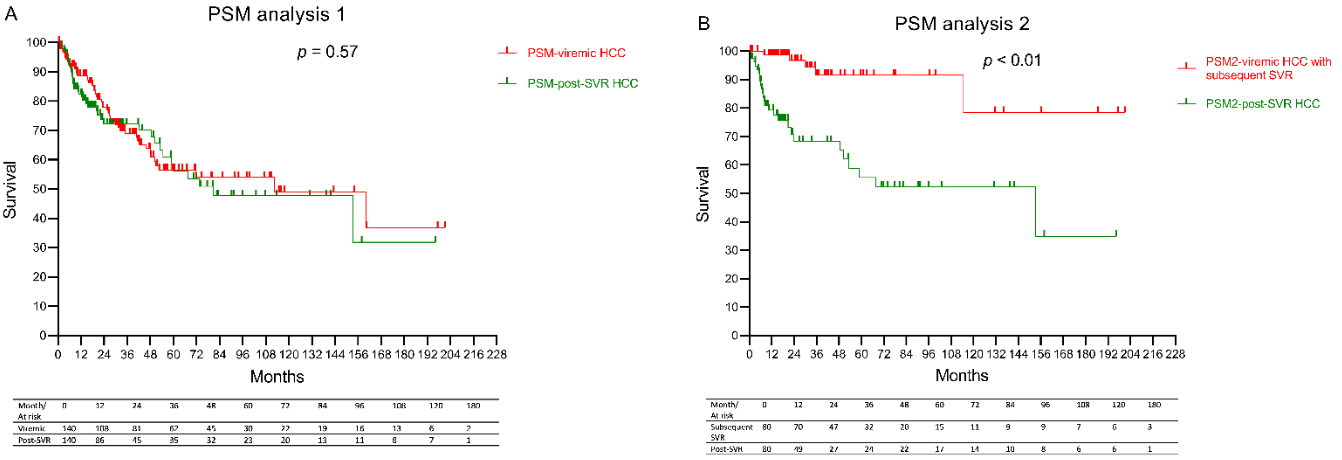

Figure S1. Overall survival of post-SVR HCC versus viremic HCC in the PSM analysis 1. (**A**), and post-SVR HCC vs. viremic HCC with subsequent SVR in the PSM analysis 2 (**B**).

Table S1. Patho-histological characteristics of resected tumor.

Tumor Characteristics	Viremic HCC *n* = 107	Post-SVR HCC *n* = 36	*p*
Pathological characters	-	-	
HCC differentiation	-	-	0.20
Well differentiation	21 (19.6)	3 (8.3)	-
Intermediate differentiation	71 (66.4)	25 (69.4)	-
Poor differentiation	15 (14.0)	8 (22.2)	-
Pathological stage	-	-	0.92
Stage 1	75 (70.1)	26 (72.2)	-
Stage 2	27 (25.2)	8 (22.2)	-
Stage 3	5 (4.7)	2 (5.6)	-
Solitary tumor	90 (84.1)	31 (86.1)	1.00
Largest tumor size, cm	2.6 (0.8–13.0)	2.7 (0.8–7.5)	0.63
Micro-vascular invasion	18 (16.8)	7 (19.4)	0.80
Fibrosis stage of non-tumor liver tissue	-	-	0.86
Stage 0	5 (5.0)	2 (5.7)	-
Stage 1	10 (9.9)	5 (14.3)	-
Stage 2	17 (16.8)	7 (20.0)	-
Stage 3	12 (11.9)	5 (14.3)	-
Stage 4	57 (56.4)	16 (45.7)	-

Continuous variables were presented with median (range), and statistics with Mann-Whitney *U* test. Categorical variables were presented with numbers (percentage), and statistics with Chi-Square and Fisher's exact test, and Pearson Chi-Square test.

Table S2. Factors associated with overall mortality in sensitivity analysis of viremic vs. post-SVR HCC excluding patients with HCC diagnosed within 12 months of SVR.

	**Univariate**		**Multivariate**	
	**HR (95% CI)**	***p***	**Adjusted HR (95% CI)**	***p***
Age, years	1.00 (0.99–1.01)	0.59	-	-
Male gender	1.26 (1.06–1.51)	0.01	1.13 (0.92–1.38)	0.26
Obesity	1.30 (1.04–1.63)	0.02	1.19 (0.95–1.50)	0.13
Alcohol	1.47 (1.24–1.75)	<0.01	1.12 (0.90–1.40)	0.30
Smoking	1.27 (1.07–1.50)	0.01	0.94 (0.77–1.15)	0.54
Metabolic disorders	1.14 (0.95–1.36)	0.16	-	-
Liver cirrhosis	2.45 (1.92–3.13)	<0.01	1.93 (1.49–2.50)	<0.01
Impaired liver status	2.17 (1.83–2.58)	<0.01	1.24 (1.00–1.53)	0.05
Platelet, × 10^3/uL	1.00 (1.00–1.00)	0.14	-	-
AST, U/L	1.00 (1.00–1.00)	<0.01	1.00 (1.00–1.00)	<0.01
ALT, U/L	1.00 (1.00–1.00)	<0.01	1.00 (1.00–1.00)	0.01
Creatinine, mg/dL	1.10 (1.04–1.17)	<0.01	1.16 (1.08–1.24)	<0.01
AFP, $\log_{10}$ ng/mL	1.72 (1.58–1.86)	<0.01	1.52 (1.40–1.65)	<0.01
BCLC 0/A (vs. B/C/D)	0.36 (0.30–0.43)	<0.01	0.57 (0.46–0.69)	<0.01
Curative therapy (vs. palliative therapy/supportive care)	0.47 (0.39–0.57)	<0.01	0.60 (0.49–0.73)	<0.01
Post-SVR HCC	1	-	1	-
Viremic HCC who remained viremic	2.21 (1.63–2.98)	<0.01	1.30 (0.92–1.84)	0.13
Viremic HCC with subsequent SVR	0.32 (0.20–0.52)	<0.01	0.16 (0.09–0.27)	<0.01

Metabolic disorder: Obesity, hypertension, or diabetes. Impaired liver status: Child-Pugh score ≥7.

Table S3. Baseline clinical and HCC characteristics at HCC diagnosis between viremic HCC and post-SVR HCC in propensity score matched analysis 1.

**Patient Characteristics**	**Viremic HCC, *n* = 140**	**Post-SVR HCC, *n* = 140**	***p***
	Clinical characteristics		
Age, years	65 (49–85)	65 (21–84)	0.40
Male gender	83 (59.3)	90 (64.3)	0.46
BMI, kg/m^2	25.0 (15.6–43.3)	25.4 (17.5–51.1)	<0.01
Obesity	23/110 (20.9)	43/119 (36.1)	0.01
Alcohol	38/134 (28.4)	43/128 (33.6)	0.42
Smoking	40/134 (29.9)	41/128 (32.0)	0.79
Hypertension	63/134 (47.0)	76/129 (58.9)	0.06
Diabetes	45/138 (32.6)	53 (37.9)	0.38
Metabolic disorders	85/137 (62.0)	97/132 (73.5)	0.05
Liver cirrhosis	101 (72.1)	100 (71.4)	1.00
Impaired liver status	22/131 (16.8)	26/137 (19.0)	0.75
Platelet, × 10^3/uL	136 (28–347)	146 (33–348)	0.21
Albumin, g/dL	3.8 (1.7–4.7)	4.1 (2.1–4.9)	<0.01
Total bilirubin, mg/dL	0.8 (0.2–3.9)	0.9 (0.2–27.7)	0.27
Prothrombin time INR	1.08 (0.89–2.40)	1.08 (0.91–2.10)	0.96
AST, U/L	40 (11–3008)	33 (18–812)	0.01
ALT, U/L	32 (6–1949)	28 (9–587)	0.06
Creatinine, mg/dL	0.9 (0.5–12.0)	0.9 (0.3–7.6)	0.19
AFP, $\log_{10}$ ng/mL	1.0 (0.3–5.1)	1.0 (−0.01–4.7)	0.65
FIB-4 index >3.25	69/134 (51.5)	65/134 (48.5)	0.71
	HCC characteristics		
BCLC stage 0/A	77 (55.0)	70 (50.0)	0.47

Solitary tumor	102/137 (74.5)	90 (64.3)	0.07
Largest tumor size, cm	2.7 (0.9–18.7)	2.4 (1.0–20.5)	0.27
Largest tumor ≥5 cm	23/137 (16.8)	19/135 (14.1)	0.62
Macroscopic vessel invasion	12 (8.6)	19/139 (13.7)	0.19
Extra-hepatic metastasis	6/128 (4.7)	7/137 (5.1)	1.00
Curative therapy	68/133 (51.1)	65/138 (47.1)	0.54

Continuous variables were presented with median (range), and statistics with Mann-Whitney *U* test. Categorical variables were presented with numbers (percentage), and statistics with Chi-Square and Fisher's exact test. Metabolic disorder: Obesity, hypertension, or diabetes. Impaired liver status: Child-Pugh score ≥7. BMI, body mass index; INR, international ratio; AST, aspartate aminotransferase, ALT, alanine aminotransferase; AFP, alpha-fetoprotein.

Table S4. Factors associated with overall mortality of viremic HCC vs. post-SVR HCC in propensity score matched analysis 1.

Variables	Univariate		Multivariate	
	HR (95% CI)	*p*	Adjusted HR (95% CI)	*p*
Age, years	1.02 (1.00–1.05)	0.10	-	-
Male gender	0.96 (0.63–1.46)	0.84	-	-
Obesity	1.32 (0.80–2.17)	0.28	-	-
Alcohol	129 (0.81–2.04)	0.28	-	-
Smoking	1.20 (0.76–1.92)	0.43	-	-
Metabolic disorders	1.28 (0.81–2.01)	0.30	-	-
Liver cirrhosis	2.31 (1.34–3.97)	<0.01	2.48 (1.37–4.48)	<0.01
Impaired liver status	2.13 (1.32–3.42)	<0.01	1.45 (0.82–2.57)	0.20
Platelet, × 10^3/uL	1.00 (1.00–1.00)	0.99	-	-
AST, U/L	1.00 (1.00–1.00)	<0.01	1.01 (1.00–1.02)	0.34
ALT, U/L	1.00 (1.00–1.00)	<0.01	0.99 (0.98–1.01)	0.42
Creatinine, mg/dL	0.89 (0.67–1.18)	0.42	-	-
AFP, $\log_{10}$ ng/mL	2.09 (1.73–2.51)	<0.01	2.21 (1.80–2.70)	<0.01
BCLC 0/A (vs. B/C/D)	0.30 (0.20–0.47)	<0.01	0.47 (0.29–0.76)	<0.01
Curative therapy (vs. palliative therapy/supportive care)	0.40 (0.25–0.62)	<0.01	0.55 (0.34–0.88)	0.01
Viremic HCC	1	-	1	-
Post-SVR HCC	1.13 (0.74–1.72)	0.57	0.93 (0.60–1.45)	0.76

Metabolic disorder: Obesity, hypertension, or diabetes. Impaired liver status: Child-Pugh score ≥7.

Table S5. Factors associated with overall mortality in post-SVR HCC and subgroups of viremic HCC patients in propensity score matched analysis 1.

	Univariate		Multivariate	
	HR (95% CI)	*p*	Adjusted HR (95% CI)	*p*
Age, years	1.02 (1.00–1.05)	0.10	-	-
Male gender	0.96 (0.63–1.46)	0.84	-	-
Obesity	1.32 (0.80–2.17)	0.28	-	-
Alcohol	129 (0.81–2.04)	0.28	-	-
Smoking	1.20 (0.76–1.92)	0.43	-	-
Metabolic disorders	1.28 (0.81–2.01)	0.30	-	-
Liver cirrhosis	2.31 (1.34–3.97)	<0.01	2.34 (1.30–4.20)	0.01
Impaired liver status	2.13 (1.32–3.42)	<0.01	1.39 (0.79–2.45)	0.26
Platelet, × 10^3/uL	1.00 (1.00–1.00)	0.99	-	-
AST, U/L	1.00 (1.00–1.00)	<0.01	1.00 (0.99–1.01)	0.47
ALT, U/L	1.00 (1.00–1.00)	<0.01	1.00 (0.98–1.01)	0.57
Creatinine, mg/dL	0.89 (0.67–1.18)	0.42	-	-
AFP, $\log_{10}$ ng/mL	2.09 (1.73–2.51)	<0.01	2.14 (1.74–2.64)	<0.01
BCLC 0/A (vs. B/C/D)	0.30 (0.20–0.47)	<0.01	0.44 (0.27–0.71)	<0.01
Curative therapy (vs. palliative therapy/supportive care)	0.40 (0.25–0.62)	<0.01	0.55 (0.34–0.88)	0.01

Post-SVR HCC	1	-	1	-
Viremic HCC who remained viremic	1.09 (0.72–1.67)	0.69	1.28 (0.82–1.98)	0.28
Viremic HCC with subsequent SVR	0.16 (0.04–0.67)	0.01	0.19 (0.04–0.81)	0.03

Metabolic disorder: Obesity, hypertension, or diabetes. Impaired liver status: Child-Pugh score ≥7.

Table S6. Baseline clinical and HCC characteristics at HCC diagnosis between viremic HCC with subsequent SVR and post-SVR HCC in propensity score matched analysis 2.

Patient Characteristics	Viremic HCC with subsequent SVR, *n* = 80	Post-SVR HCC, *n* = 80	*p*
Clinical characteristics			
Age, years	64 (47–89)	62 (21–82)	0.31
Male gender	45 (56.3)	55 (68.8)	0.14
BMI, kg/m^2	25.0 (16.5–41.1)	25.9 (20.4–51.1)	<0.01
Obesity	16/56 (28.6)	31/69 (44.9)	0.07
Alcohol	15/61 (24.6)	32/75 (42.7)	0.03
Smoking	19/61 (31.1)	28/75 (37.3)	0.47
Hypertension	31/61 (50.8)	45/75 (60.0)	0.30
Diabetes	24/77 (31.2)	31 (38.8)	0.40
Metabolic disorders	47/67 (70.1)	58/78 (74.4)	0.58
Liver cirrhosis	60 (75.0)	65 (81.3)	0.45
Impaired liver status	16 (20.0)	24 (30.0)	0.20
Platelet, × 10^3/uL	138 (5–688)	144 (38–332)	0.13
Albumin, g/dL	3.8 (1.7–4.9)	4.0 (2.1–4.9)	0.10
Total bilirubin, mg/dL	0.9 (0.2–5.7)	1.0 (0.2–27.7)	0.39
Prothrombin time INR	1.09 (0.95–1.70)	1.10 (0.95–2.10)	0.84
AST, U/L	51 (19–135)	43 (18–812)	0.05
ALT, U/L	38 (18–186)	36 (16–587)	0.76
Creatinine, mg/dL	0.8 (0.5–6.5)	1.0 (0.3–7.6)	0.09
AFP, $\log_{10}$ ng/mL	1.2 (0.4–4.5)	1.1 (−0.01–4.6)	0.90
FIB-4 index >3.25	48 (60.0)	43/78 (55.1)	0.63
HCC characteristics			
BCLC stage 0/A	46/79 (58.2)	43 (53.8)	0.63
Solitary tumor	51/74 (68.9)	50 (62.5)	0.50
Largest tumor size, cm	2.2 (0.9–7.5)	2.4 (1.0–20.5)	0.26
Largest tumor ≥5 cm	7/79 (8.9)	12/77 (15.6)	0.23
Macroscopic vessel invasion	4 (5.0)	12 (15.0)	0.06
Extra-hepatic metastasis	0/77 (0)	5/78 (6.4)	0.06
Curative therapy	39/77 (50.6)	35/78 (44.9)	0.52

Continuous variables were presented with median (range), and statistics with Mann-Whitney *U* test. Categorical variables were presented with numbers (percentage), and statistics with Chi-Square and Fisher's exact test. Metabolic disorder: Obesity, hypertension, or diabetes. Impaired liver status: Child-Pugh score ≥7. BMI, body mass index; INR, international ratio; AST, aspartate aminotransferase, ALT, alanine aminotransferase; AFP, alpha-fetoprotein.

Table S7. Factors associated with overall mortality of viremic HCC with subsequent SVR vs. post-SVR HCC in propensity score matched analysis 2.

	Univariate		Multivariate	
	**HR (95% CI)**	***p***	**Adjusted HR (95% CI)**	***p***
Age, years	1.01 (0.97–1.05)	0.72	-	-
Male gender	0.99 (0.48–2.08)	0.99	-	-
Obesity	1.65 (0.72–3.75)	0.23	-	-
Alcohol	1.18 (0.54–2.59)	0.69	-	-
Smoking	1.00 (0.45–2.25)	1.00	-	-
Metabolic disorders	1.85 (0.70–4.88)	0.21	-	-
Liver cirrhosis	4.60 (1.10–19.30)	0.04	1.64 (0.36–7.46)	0.53
Impaired liver status	2.52 (1.23–5.19)	0.01	1.71 (0.76–3.85)	0.19
Platelet, × 10^3/ul	1.00 (0.99–1.00)	0.55	-	-
AST, U/L	1.00 (1.00–1.01)	0.33	-	-
ALT, U/L	1.00 (1.00–1.01)	0.68	-	-
Creatinine, mg/dl	1.05 (0.58–1.92)	0.87	-	-
AFP, log_{10} ng/ml	2.30 (1.63–3.25)	<0.01	2.38 (1.65–3.41)	<0.01
BCLC 0/A (vs. B/C/D)	0.21 (0.09–0.46)	<0.01	0.27 (0.11–0.62)	<0.01
Curative therapy (vs. palliative therapy/supportive care)	0.22 (0.09–0.55)	<0.01	0.33 (0.13–0.82)	0.02
Post-SVR HCC	1	-	1	-
Viremic HCC with subsequent SVR	0.16 (0.06–0.41)	<0.01	0.15 (0.05–0.39)	<0.01

Metabolic disorder: Obesity, hypertension, or diabetes. Impaired liver status: Child-Pugh score ≥7.
